# Dynamics of photosynthetic responses in 10 rubber tree (*Hevea brasiliensis*) clones in Colombian Amazon: Implications for breeding strategies

**DOI:** 10.1371/journal.pone.0226254

**Published:** 2019-12-12

**Authors:** Armando Sterling, Natalia Rodríguez, Esther Quiceno, Faiver Trujillo, Andrés Clavijo, Juan Carlos Suárez-Salazar

**Affiliations:** 1 Laboratorio de Fitopatología, Instituto Amazónico de Investigaciones Científicas Sinchi–Facultad de Ciencias Básicas—Universidad de la Amazonía, Florencia, Colombia; 2 Laboratorio de Ecofisiología, Universidad de la Amazonia, Facultad de Ingeniería, Programa de Ingeniería Agroecológica, Florencia-Caquetá, Colombia; United Arab Emirates University, UNITED ARAB EMIRATES

## Abstract

The rubber tree [*Hevea brasiliensis* (Willd. Ex Adr. de Juss.) Muell.-Arg] is the main source of natural rubber in the world. However, in the Amazon region, its production is reduced by biotic and abiotic limitations, which have prompted breeding programs in order to identify desirable agronomic and physiological indicators. The objective of this study was to analyze the temporal dynamics of photosynthetic responses based on the parameters of leaf gas exchange and chlorophyll *a* fluorescence in 10 rubber tree clones during the immature phase (pre-tapping) in three large-scale clone trials, during daily cycles and under two climatic periods (dry and rainy) in the Caquetá region (Colombian Amazon). The variables *A*, *LT*, *Φ*_PSII_, *ETR* and *qP* were significantly higher in the dry period, where the highest values of *PAR*, *AT* and *VPD* were seen. In San Vicente del Caguán and Florencia, the highest averages were estimated for *A*, *E* and *g*_*s*_, as compared with Belén de los Andaquíes. In Florencia, the highest fluorescence parameters of chlorophyll *a* were recorded. At 9:00 h and 12:00 h, the highest means of *A*, *E*, *Φ*_PSII_ and *ETR* were observed. The majority of the clones displayed the highest *F*_*v*_*/F*_*m*_ mean (0.82–0.84) in the dry period. The clones FX 4098, FDR 4575, MDF 180, GU198 and FDR 5788 represent genotypes with the best photosynthetic performance (greater photosynthetic rates and better ability of the photosynthetic apparatus to capture, use and dissipate light energy). These desirable genotypes constitute a promising gene pool for expanding the genetic resource of rubber trees in the Colombian Amazon.

## Introduction

The rubber tree [*Hevea brasiliensis* (Willd. Ex Adr. de Juss.) Muell.-Arg] is a South American (Amazon region) native species and is the most important natural rubber source globally [1]. Worldwide, the Asia-pacific region produces 91.2% of natural rubber, Africa produces 6.8% and Central and South America produce 2.0% [2].

Breeding programs for this species have used domestication as the main strategy in order to generate genotypes with high productive performance and tolerance to the principal biotic and abiotic limitations of crops [3]. In this sense, various efforts have been made to select more productive varieties that adapt to different agro-climatic conditions, based on the evaluation of agronomic parameters related to yield, biomass production, water use efficiency and diseases resistance [4–10].

In Colombia, in 2008, SINCHI (Amazonian Institute of Scientific Research), the University of the Amazon and the Association of Rubber Reforesters and Cultivators of Caquetá (ASOHECA) began expanding the genetic resources of *H*. *brasiliensis* in Caquetá (Amazon region) by evaluating American-origin clones in large-scale clone trials [11], in order test growth, nutritional behavior, reaction to diseases and pests, phenology and, lastly, to assess latex production (tapping) over a period of 5–10 years.

However, an important aspect in rubber breeding programs is the selection of genotypes resistant to environmental variations by monitoring the physiological response of plants under *in vivo* conditions and evaluating the ability of plants to survive in adverse environmental conditions [12–14]. Knowledge on this response will be useful for understanding the adaptive potential of these rubber clones in different environments, minimizing downtime and maximizing productive performance.

In the genetic breeding programs of various crops, a method of rapid and early selection of genotypes with desirable characteristics, such as high yield or tolerance to environmental stress, has been implemented based on the analysis of physiological characteristics [10] as reported in *Gmelina arborea* Roxb. [15], *Coffea* spp. [16], *Eucalyptus* spp. [17], *Pinus* spp. [18] and *Populus* spp. [19].

The most commonly used physiological parameters include relationships with photosynthesis because of the immediate response of the photosynthetic apparatus to most biotic and abiotic factors that can cause stress conditions [20]. According to Holá et al. [20], there are three categories of photosynthetic parameters recommended in breeding programs: 1) variables associated with gas exchange, 2) content of photosynthetic pigments (e.g. chlorophyll *a* and *b*, carotenoids) and 3) chlorophyll *a* fluorescence. Brestic et al. [21] mentioned that the gas exchange measurements are supported by continuous measurements of chlorophyll fluorescence that provide quite a precise estimation of photosynthetic performance, and chlorophyll fluorescence represents a unique tool for diagnostics of plant health status, photosynthetic performance as well as effects of plant stress on plants and assessment of plant stress tolerance [22,23].

The photosynthesis is the key process necessary for plant production. Through the process of photosynthesis of C3 plants is the net result of concurrent processes in which light energy is used to produce ATP and NADPH in the light reaction [24] and subsequently, CO_2_ is fixed (carboxylation) and released (photorespiration, day respiration) [25]. And the gas exchange measurements are essential for the characterization of leaf photosynthetic properties, including stomatal conductance, carboxylation rate, or water use efficiency [25]. Since rubber is a perennial species that requires more than six years of growth before latex can be harvested (unproductive period) and another seven years to assess its potential yield in the productive period [26], it is essential to analyze this type of physiological indicator, which facilitates the identification of the adaptive potential of new genetic materials in the face of various environmental conditions and, thus, optimizes the growth phase in order to reduce the time required to start the productive phase.

Since field conditions expose rubber trees to various environmental variations throughout the day and at different times of the year, in this study hypothesized that these variations have differential effects on the photosynthetic performance of *H*. *brasiliensis* genotypes with different adaptive capacities. To test this hypothesis, the objective of this study was to analyze the temporal dynamics of photosynthetic responses based on the parameters of leaf gas exchange and chlorophyll *a* fluorescence in nine promising American clones and the IAN 873 clone (control) during the immature phase (pre-tapping) in large-scale clone trials as a measurement of the clones’ specific adaptation to agro-climatic conditions in the Colombian Amazon.

## Materials and methods

### Study area

#### Experimental sites

The experiments were established in July, 2009 on threes farms owned by rubber producers of the Association of Rubber Reforesters and Cultivators of Caquetá (ASOHECA) in the Department of Caquetá (Colombian Amazon), on land with a moderately undulating topography and edaphoclimatic variations. The study area was had compact soils with very low fertility, high acidity and high aluminum contents as a result of being abandoned pastures (> 20 years land use) with an extensive livestock farming traditional in the region. The first experiment was established in Belén de los Andaquíes (1°25'28'' north and 75°52'11'' west, at an elevation of 300 m above sea level). The second experiment was established in Florencia (1°37'03'' north and 75°37'03'' west, at an elevation of 270 m above sea level). The third experiment was established in San Vicente del Caguán (2°2'40.8'' north and 74°55'11.7'' west, at an elevation of 344 m above sea level).

#### Climate

Caquetá is a humid tropical region that spans two hemispheres, with almost vertical solar radiation during the entire year [27]. Caquetá has a monomodal climatic regime [28,29], subdivided into the “ecological summer” period that corresponds to the months of November to February (dry period) and a “ecological winter” period that corresponds to the months of March to June (rainy period), the other months correspond to an interval close to the average of the precipitation volumes [30].

According to the Caldas-Lang climate classification [27], Belén de los Andaquíes and Florencia have a warm-humid climate, while San Vicente del Caguán is a warm-semi-humid climate. Florencia has an average temperature of 25ºC, an average relative humidity of 84%, a solar brightness of 1,465.4 h light and a precipitation of 3,669 mm year^-1^. Belén de los Andaquíes has an average temperature of 25ºC, an average relative humidity of 85.7%, a solar brightness of 1,462.3 h light and a rainfall of 3,471 mm year^-1^. San Vicente del Caguán has an average temperature of 25.4ºC, an average relative humidity of 79%, a solar brightness of 1,552.3 h light and a rainfall of 2,503 mm year^-1^.

**Fig 1:**
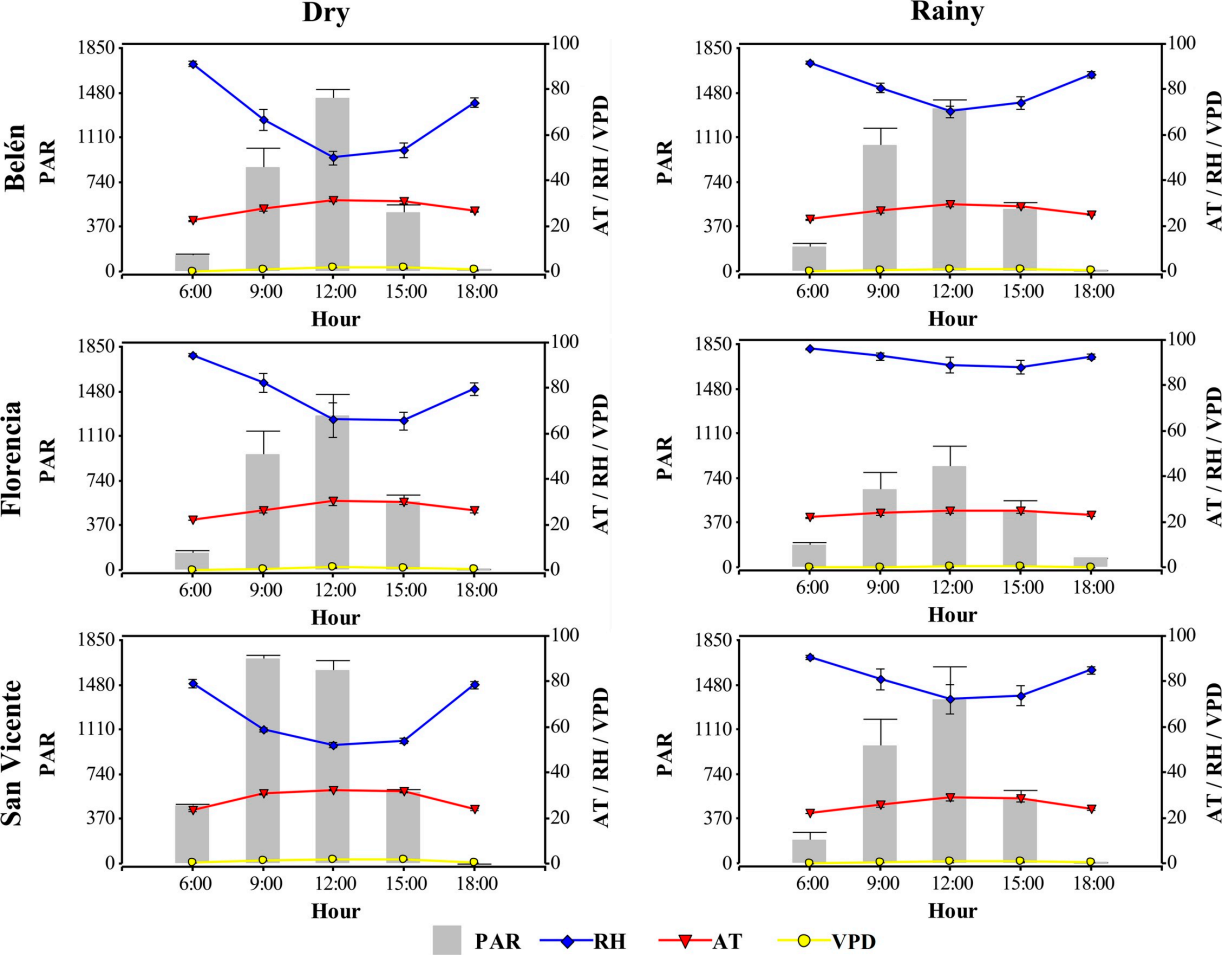
Daily microclimatic variation in study area (Caquetá, Colombia). Data averages at Belén de los Andaquíes, Florencia and San Vicente del Caguán in two climatic period: dry (November 2017 to January 2018) and rainy (May to June 2018). Photosynthetically active radiation (*PAR*), relative humidity (*RH*), atmosphere temperature (*AT*) and vapor pressure deficit (*VPD*). The values represent the mean, and the bars the standard error (*n* = 60).

The microclimatic factors: photosynthetically active radiation (*PAR*), relative humidity (*RH*), air temperature (*AT*) and vapor pressure deficit (*VPD*) were provided by a SINCHI weather station (Amazonian Institute of Scientific Research) in each experimental site. The data on an average day at 6:00, 9:00, 12:00, 15:00 and 18:000 h was calculated in two climatic period (dry and rainy) for *PAR*, *RH*, *AT* and *VPD* (Fig 1).

#### Soils

Caquetá’s soils are poorly drained, very superficial and deep, with a high aluminum saturation and low base saturation; it has a low quantity of Calcium, Magnesium, Potassium, Phosphorus and Sodium [27]. Belén de los Andaquíes has soils with a pH of 4.8, very acidic, with an organic matter content of 1.07%, organic carbon content of 0.62%, median saturation of 27.7%, and a clay texture. Florencia has soils with a pH of 4.85, very acidic, with an organic matter content of 0.92%, organic carbon content of 0.54%, median saturation of 29.58%, and a clay texture. San Vicente del Caguán has soils with a pH of 4.89, very acidic, with an organic matter content of 0.91%, organic carbon content of 0.60%, median saturation of 28.75%, and a clay-sandy loam texture.

### Plant material

The 10 rubber clones used in these trials were *H*. *brasiliensis* clones from Central and South America (Table 1) and were chosen because of their good phytosanitary, vigor and production characteristics, mostly achieved on an experimental scale (plant breeding) in countries such as Brazil [31–33] and Ecuador [34]. Clone IAN 873 was chosen as the control since it is one of the most widely planted in countries such as Colombia [35].

**Table 1:** Plant material description. The ten rubber tree clones tested in Florencia, Belén de los Andaquíes and San Vicente del Caguán, Caquetá (Colombia), 2017–2018.

Clone	Parents (Female x Male)	Country of origin	Year of introduction into Caquetá (Colombia)
CDC 56	MDX 91 x RRIM 614	Guatemala	2002
CDC 312	AVROS 308 x MDX 40	Guatemala	2002
GU 198	GT 711 x FX 16	Guatemala	2000
IAN 873 (control)	PB 86 x FA 1717	Brazil	1964
FX 4098	PB 86 x B 110	Brazil	2000
FX 3899 P1 (polyploid)	F4542 x AVROS 363	Brazil	1996
MDF 180	Primary clone	Peru	2002
FDR 4575	FDR 18 x FX 3032	Brazil	2002
FDR 5597	HAR 68 x TU 42–525	Brazil	2002
FDR 5788	HAR 8 x MDF 180	Brazil	2002

AVROS: Algemene Vereniging Rubberplanters Oostkust Sumatra; B: Belterra, Brazil; CDC: Clavellinas Dothidella Cross; FB: Ford Belem; FDR: Firestone Dothidella Resistant; FX: Ford Cross; MDF: Madre de Dios Firestone; MDX: Madre de Dios Cross; HAR: Harbel Estate (Firestone), Liberia; TU: Turrialba, Costa Rica; PB: Prang Besar, Malaysia; GT: Gondang Tapen, Indonesia; FA: Ford Acre; F: Ford, Brasil; GU: Guatemala; IAN: Instituto Agronômico do Norte; RRIM: Rubber Research Institute of Malaysia

### Experimental design and maintenance of the plots

A large-scale clonal trail (LSCT) was established in each site [36]. Each LSCT followed a randomized complete block design with 10 treatments (genotypes) and four replications randomly arranged in Fisher blocks, with 60 trees per replicate and per clone. The planting distance was 7.0 m × 3.0 m, providing a density of 476 trees per hectare, for a total LSCT area of 5.04 ha. The unit plot was 1,260 m^2^, corresponding to 60 trees organized in 3 rows of 20 trees. The trial was surrounded by one row of Colombian mahogany (*Cariniana pyriformis* Miers) as a windbreaker barrier.

Each plot employed fertilization management with a frequency every of six months using a compound fertilizer [N (15%), P_2_O_5_ (15%), K_2_O (15%), CaO (2.2%), S-SO_4_ (1.7%)] with a dosage of 150 g plant^-1^, a fertilizer with minor elements [N (8%), P_2_O_5_ (5%), CaO (18%), MgO (6%), S (1.6%), B (1%), Cu (0.14%), Mo (0.005%) and Zn (2.5%)] (75 g plant^-1^) and organic matter (1,000 g plant^-1^). Weeds were removed with mechanical controls with a frequency of every three months. Phytosanitary controls were not carried out.

### Photosynthetic and micro-environmental parameters

The photosynthetic and micro-environmental parameters at the leaf level were measured in each site for two climatic periods: a) dry period: December 13–17, 2017 in San Vicente del Caguán; January 12–16, 2018 in Belén de los Andaquíes; January 16–20, 2018 in Florencia. b) rainy period: May 10–14, 2018 in San Vicente del Caguán; May 19–24, 2018 in Belén de los Andaquíes; June 6–10, 2018 in Florencia.

The photosynthetic light response curves (*A/PAR*), leaf gas exchange, micro-environmental parameters at the leaf level and chlorophyll *a* fluorescence were measured with a portable photosynthesis system (CIRAS-3 PP Systems, USA) coupled with a chlorophyll fluorescence module (CFM-3 PP Systems, Amsbury, MA, USA). The CO_2_ flow was maintained at a concentration of 390 μmol mol^-1^, with a cuvette temperature (*CT*) of 27°C, an average relative humidity (*RH*) of 70% and a vapor pressure deficit (*VPD*) 2.5 kPa on average.

The photosynthetic light responses curves (*A/PAR*) were done per clone in order to determine the constant value of *PAR* to be used in all measurements with CIRAS-3 in all clones for all three experimental sites (*PAR* = 1,067 μmol photons m^-2^ s^-1^). The *PAR* intensity was modulated in decreasing order in 16 steps between 2,500 to 0 μmol photons m^-2^s^-1^, between 9:00 to 12:00 h. The parameters derived from the *A/PAR* curve included the maximum photosynthetic rate at saturating light (*A*_*max*_), the light compensation point (*LCP*), the light saturation point (*LSP*), the dark breathing rate (*R*_*d*_), and the apparent quantum efficiency (*A*_*qe*_), which were fit to the Mitscherlich Model [37].

The gas exchange variables were measured at the foliar level: net photosynthesis rate (*A*) (μmol CO_2_ m^-2^ s^-1^), transpiration rate (*E*) (mmol H_2_O m^-2^ s^-1^), stomatal conductance (*g*_*s*_) (mmol H_2_O m^-2^ s^-1^), concentration of intercellular CO_2_ (*C*_*i*_) and leaf temperature (*LT*) [38], as well as the micro-environmental parameters *PAR*, *RH*, *VPD y AT*. The measurements were taken in a daily cycle between 6:00 h and 18:00 h at 3-hour intervals on sunny days. Two healthy leaves with physiological maturity in foliar stage D (140–150 days old) were selected [39], which were fully expanded and found in the middle-third of the canopy, in four trees per clone. The chlorophyll content index (*CCI*) was also measured as a leaf selection criterion, defining a range between 18 to 38 units in the central leaflet of each leaf. The *CCI* was measured with a chlorophyll concentration meter (MC-100, Apogee Instruments Inc., USA).

The chlorophyll *a* fluorescence measurement was recorded simultaneously with the same leaves used for the gas exchange parameters. A predawn measurement (3:00 h) was taken to ensure that the leaves were adapted to darkness. Following this period of adaptation to darkness, the leaf tissue was exposed to a weak modulated pulse (0.03 μmol m^-2^ s^-1^, non-actinic) to obtain the minimum fluorescence (*F*_*o*_). A pulse of white saturating light (6,000 μmol m^-2^ s^-1^) was then emitted for 1 s to obtain the maximum fluorescence (*F*_*m*_) and, thus, calculate the maximum photochemical efficiency of PSII (*F*_*v*_*/F*_*m*_) [40]. Subsequently, a pulse of actinic light (9,000 μmol photons m^-2^ s^-1^) was used to measure the steady state fluorescence yield (*F*_*s*_) and the maximum light adapted fluorescence (*F*_*m*_*'*). Once the actinic light was removed, the leaf was exposed to a pulse of far red light in order to oxidize the quinone *Q*_*A*_ to the maximum and estimate the minimum fluorescence in light-adapted leaves (*F*_*o*_*'*), guaranteeing that the PSII reaction centers opened again. The parameters of chlorophyll fluorescence estimated in dark-adapted leaves were:

The efficiency of excitation energy captured by open PSII reaction centers (*F*_*v*_*'/F*_*m*_*'*), can be used to provide an estimate of the maximum efficiency of PSII photochemistry in the light-adapted state [23], and was calculated as:

$$F_{v}\prime /F_{m}\prime =F_{m}\prime -F_{o}\prime /F_{m}\prime$$ (1)

The parameter *qP* gives an indication of the proportion of PSII reaction centers that are already open [41]. This was calculated as:

$$qP=(F_{m}\prime -F_{s})/(F_{m}-F_{o}\prime )$$ (2)

To evaluate the changes in the apparent rate constant for excitation decay by heat loss induced by light relative to this constant rate in the dark, the parameter *NPQ* was assessed [40]. This parameter was calculated as:

$$NPQ=(F_{m}-F_{m}\prime )/F_{m}\prime$$ (3)

The parameters of chlorophyll *a* fluorescence estimated in light-adapted leaves in the daily cycle (6:00 to 18:00 h) were:

The photochemical efficiency of PSII (*Φ*_PSII_) measures the proportion of the light that is absorbed by chlorophyll associated with PSII and is used in photochemistry [42]; it was calculated as:

$$\Phi_{PSII}=(F_{m}\prime -F_{t})/F_{m}\prime$$ (4)

The apparent electron transport rate (*ETR*), which is an indicator of overall photosynthetic capacity *in vivo*, was determined as follows [41]:

$$ETR=PAR\;x\;0.84\;x\;0.5\;x\;\Phi_{PSII}$$ (5)

where *PAR* is absorbed light, and 0.50 is the factor that accounts for the partitioning of energy.

### Data analysis

A mixed general linear model (MGLM) was adjusted to analyze the effect of the fixed factors (sources of variation): climatic period, site, clone, hour and their interactions on the physiological variables. The assumptions of the GLM (normality and homogeneity of variance) were evaluated using an exploratory residual analysis. The nested blocks in the sites and the plots associated with the genotypes within the blocks were included as random effects. The residual variance was modeled to contemplate different variances (Heteroscedasticity), while the residual correlation for the successive observations (hour) carried out on the same plant was contemplated with the models generally used for longitudinal data. Akaike (AIC), Bayesian (BIC) and Log lik criteria were used to select the structure of residual variances and correlations [43]. The analyses were carried out using the *lme* function in the *nlme* package [44] in R language software, version 3.4.1 [45], and the interface in InfoStat v. 2017 [46]. Differences between mean variables in all fixed factors were analyzed with Fisher’s LSD post-hoc test at a significance of α = 0.05. The coefficients of correlation (Pearson’s test) between the physiological variables were estimated for each of the periods.

To explore the relationships between the physiological variables and the microclimatic parameters associated with 10 rubber tree clones, a Co-Inertia analysis was carried out on the covariance matrix [47]. A Monte Carlo test was carried out to determine the significance of the Co-Inertia values, using ADE-4 [48], included in the R 3.4.1 package [45].

## Results

### Diurnal changes in the microclimatic factors

According to Fig 1, the *PAR* (682 μmol photon m^-2^ s^-1^), *AT* (28ºC) and *VPD* (1.0 kPa) were always highest in the dry period. The highest values of *PAR* and *AT* were observed from 9:00 to 12:00 h in San Vicente del Caguán (1,600 μmol photon m^-2^ s^-1^ and 32ºC, respectively). In Belén de los Andaquíes and Florencia, the highest *PAR* values were recorded at 12:00 h with 1,434 and 1,274 μmol photon m^-2^ s^-1^, respectively. The higher *VPD* values were observed from 12:00 to 15:00 h (1.6 kPa) in San Vicente del Caguán and Belén de los Andaquíes. The *RH* acted contrary to the *PAR*, with the minimum values generally at 12:00 and 15:00 h, below 60%; San Vicente del Caguán recorded the lowest values in this period climatic.

Meanwhile, the rainy period reached the maximum *RH*, with average of 86%, but had the lowest values of *AT* (26ºC), *PAR* (558 μmol photon m^-2^ s^-1^) and *DPV* (0.43 kPa). The maximum *RH* values were recorded at 6:00 and 18:00 h in Florencia, above 95%, and the lowest value was in Belén de los Andaquíes and San Vicente del Caguán at 12:00 and 15:00 h with 75%.

### Photosynthetic light response

Significant differences in the photosynthetic response to light were observed between the 10 clones (Table 2). Clone CDC 56 presented the highest *A*_*max*_, while clone FDR 5788 had a 21.9% lower CO_2_ assimilation rate. However, this higher carbon fixation was not always related to greater efficiency of the photosynthetic apparatus (*A*_*qe*_), as occurred in clone GU 198. Clone FDR 5597 had the highest *LCP* value, double that registered for clone FDR 4575, which had the lowest value. The *LSP* in most of the clones was above 1,000 μmol photons m^-2^s^-1^, where clone FDR 5597 had the highest value, while GU 198 presented the lowest saturation point. As for *R*_*d*_, clone FDR 4575 presented the lowest value in this process, while clone FX 3899 P1 had 63% higher substrate consumption.

**Table 2:** Parameters derived from the photosynthetic light (*A/PAR*) response curves of 10 rubber tree (*Hevea brasiliensis*) clones in Colombian amazon. *A*_*max*_, light-saturated net carbon assimilation rate; *LCP*, light compensation point; *LSP*, light saturation point; *R*_*d*_, dark respiration rate; *A*_*qe*_, quantum efficiency. Data are shown as mean value ± SE (n = 4).

Clone	*A*_*max*_ (μmol m-^2^ s-^1^)	*LCP* (μmol m-^2^ s-^1^)	*LSP* (μmol m-^2^ s-^1^)	*R*_*d*_ (μmol m-^2^ s-^1^)	*A*_*qe*_^a^ (μmol CO_2_ μmol photons-^1^)
CDC 312	12.89 ± 0.44^b^b^c^	94.52 ± 9.50 a	1160.52 ± 28.76 c	-2.92 ± 0.35 a	2.2 ± 0.2 c
CDC 56	15.03 ± 0.25 a	74.13 ± 4.62 b	1225.41 ± 13.19 b	-2.47 ± 0.19 a	2.1 ± 0.1 c
FDR 4575	14.97 ± 0.35 a	52.91 ± 7.03 c	1054.02 ± 19.62 e	-1.99 ± 0.32 b	2.4 ± 0.2 c
FDR 5597	13.11 ± 0.51 b	109.23 ± 10.28 a	1321.11 ± 30.87 a	-3.04 ± 0.34 a	1.9 ± 0.2 c
FDR 5788	11.73 ± 0.18 c	96.10 ± 4.29 a	1097.21 ± 12.85 d	-2.94 ± 0.16 a	2.3 ± 0.1 c
FX 3899 P1	13.62 ± 0.42 b	91.67 ± 8.77 a	1138.30 ± 25.96 c	-3.15 ± 0.37 a	2.2 ± 0.2 c
FX 4098	13.09 ± 0.39 b	60.09 ± 8.19 c	854.08 ± 25.21 f	-2.54 ± 0.43 b	3.0 ± 0.3 b
GU 198	12.67 ± 0.26 b	53.23 ± 5.60 c	772.79 ± 16.85 h	-2.33 ± 0.30 b	3.2 ± 0.3 a
IAN 873	12.75 ± 0.34 b	93.64 ± 7.35 a	1140.27 ± 22.05 c	-2.91 ± 0.28 a	2.2 ± 0.2 c
MDF 180	12.95 ± 0.15 b	55.98 ± 3.30 c	908.79 ± 9.59 g	-2.15 ± 0.16 b	2.7 ± 0.1 b

^a^1x10^-3^

^b^Standard error

^c^Values in each column followed by the same letter do not differ statistically (Fisher’s LSD test, *p* < 0.05)

### Foliar micro-environmental parameters

The micro-environmental parameters measured at the foliar level showed that in dry period recorded the highest average values of *PAR* (258 μmol m^-2^ s^-1^), *AT* (33ºC) and *VPD* (2.6 kPa). The rainy period recorded the highest average values of *RH*, with 50%. The maximum values of *PAR* (560 μmol m^-2^ s^-1^), *RH* (54%) and *AT* (32ºC) were obtained in San Vicente del Caguán in both climatic periods, while the *VPD* (2.9 kPa) was higher in Belén de los Andaquíes in the dry period. At the three sites, the maximum *PAR* (480 μmol m^-2^ s^-1^) occurred at midday in the dry period, as compared with the rainy period (430 μmol m^-2^ s^-1^). This diurnal pattern for maximum values at midday was also observed for *VPD* and *AT*.

### Gas exchange and chlorophyll fluorescence *a*

Significant effects were observed from all of the principal effects on the gas exchange variables (with the exception of *LT*) and the fluorescence parameters of chlorophyll *a* (Table 3). There were significant differences in the higher order interaction for all the gas exchange variables, with the exception of the *Φ*_PSII_ and *ETR* variables. The parameters *F*_*v*_*/F*_*m*_, *F*_*v*_***'****/F*_*m*_***'*** and *qP* showed significant differences between the periods, between the sites and in the interaction between both factors. *qP* presented significant differences in the interaction between the factors period, site and clone.

**Table 3:** Analysis of variance of the fixed effects. Period (P), site (S) clone (C), hour (H), and their interactions, on the net CO_2_ assimilation rate (*A*), transpiration rate (*E*), stomatal conductance (*g*_*s*_), intercellular CO_2_ concentration (*C*_*i*_), leaf temperature (*LT*), photochemical efficiency of PSII (*Φ*_PSII_), electron transport rate (*ETR*), maximum photochemical efficiency of PSII (*F*_*v*_*/F*_*m*_), efficiency of excitation energy captured by open PSII reaction centers (*F*_*v*_*'/F*_*m*_*'*), photochemical quenching coefficient (*qP*), and non-photochemical quenching (*NPQ*).

Variables	*F* based *P* values										
	*A*	*E*	*g*_*s*_	*C*_*i*_	*LT*	Φ_PSII_	ETR	*F*_*v*_*/F*_*m*_	*F*_*v*_*'/F*_*m*_*'*	*qP*	*NPQ*
**P**	<0.0001	0.0001	<0.0001	<0.0001	0.1645	0.0097	0.0009	<0.0001	<0.0001	<0.0001	0.5387
**S**	0.0004	0.0003	0.0009	0.0002	<0.0001	<0.0001	<0.0001	<0.0001	<0.0001	0.0102	0.5893
**C**	<0.0001	<0.0001	<0.0001	0.0001	<0.0001	<0.0001	<0.0001	0.1284	0.4457	0.0567	0.6490
**H**	<0.0001	<0.0001	<0.0001	<0.0001	<0.0001	<0.0001	<0.0001	-	-	-	-
**P x S**	0.0012	<0.0001	<0.0001	<0.0001	<0.0001	<0.0001	<0.0001	<0.0001	<0.0001	0.0220	0.2861
**P x C**	<0.0001	<0.0001	<0.0001	0.2292	<0.0001	0.0052	0.0064	0.1705	0.0750	0.0017	0.1034
**P x H**	<0.0001	<0.0001	<0.0001	<0.0001	<0.0001	<0.0001	<0.0001	-	-	-	-
**S x C**	<0.0001	<0.0001	<0.0001	0.0079	<0.0001	<0.0001	<0.0001	0.1507	0.2941	0.3902	0.8141
**S x H**	<0.0001	<0.0001	<0.0001	<0.0001	<0.0001	<0.0001	<0.0001	-	-	-	-
**C x H**	<0.0001	<0.0001	<0.0001	<0.0001	<0.0001	0.0531	0.0473	-	-	-	-
**P x S x C**	<0.0001	<0.0001	<0.0001	0.0280	<0.0001	0.0008	0.0009	0.0420	0.0732	0.0021	0.8236
**P x S x H**	<0.0001	<0.0001	<0.0001	<0.0001	<0.0001	<0.0001	<0.0001	-	-	-	-
**P x C x H**	0.0057	0.1495	0.0025	<0.0001	<0.0001	0.1438	0.1550	-	-	-	-
**S x C x H**	<0.0001	<0.0001	<0.0001	<0.0001	<0.0001	0.0003	0.0002	-	-	-	-
**P x S x C x H**	0.0089	0.0271	0.0124	<0.0001	<0.0001	0.0670	0.0668	-	-	-	-

- Does not apply

Most of the photosynthetic parameters were significantly higher in the rainy period, except for *A*, *LT*, *Φ*_*PSII*_, *ETR* and *qP*, with higher means in the dry period (Table 4). In San Vicente del Caguán and Florencia, the highest averages were estimated for *A*, *E* and *g*_*s*_, as compared with Belén de los Andaquíes (Table 4). In Florencia, the highest fluorescence parameters of chlorophyll *a* were recorded.

**Table 4:** Mean values for the leaf gas exchange and fluorescence parameters of chlorophyll *a*. Net CO_2_ assimilation rate (*A*), transpiration rate (*E*), stomatal conductance (*g*_*s*_), intercellular CO_2_ concentration (*C*_*i*_), leaf temperature (*LT*), photochemical efficiency of PSII (*Φ*_PSII_), electron transport rate (*ETR*), maximum photochemical efficiency of PSII (*F*_*v*_*/F*_*m*_), efficiency of excitation energy captured by open PSII reaction centers (*F*_*v*_*'/F*_*m*_*'*) and photochemical (*qP*) and non-photochemical quenching coefficients (*NPQ*).

Factor	Level	Variables										
		*A*	*E*	*g*_*s*_	*Ci*	*LT*	*Φ*_PSII_	*ETR*	*Fv/Fm*	*Fv´/Fm´*	*qP*	*NPQ*
	**Rainy**	5.21 ± 0.168^a^ b^b^	3.14 ± 0.064 a	174.91 ± 5.278 a	319.46 ± 1.52 a	32.65 ± 0.046 b	0.22 ± 0.003 b	96.41 ± 1.215 b	0.826 ± 0.001 a	0.82 ± 0.001 a	0.75 ± 0.006 b	0.02 ± 0.003 a
**Period**	**Dry**	5.90 ± 0.168 a	2.89 ± 0.064 b	133.05 ± 5.278 b	301.90 ± 1.52 b	32.74 ± 0.046 a	0.23 ± 0.003 a	100.39 ± 1.215 a	0.801 ± 0.001 b	0.80 ± 0.001 b	0.81 ± 0.006 a	0.02 ± 0.003 a
												
	**Belen**	4.20 ± 0.256 b	2.48 ± 0.097 b	116.59 ± 7.892 b	318.34 ± 2.02 a	33.28 ± 0.062 a	0.22 ± 0.004 b	100.05 ± 1.628 b	0.825 ± 0.002 b	0.82 ± 0.002 b	0.78 ± 0.008 a	0.02 ± 0.004 a
**Site**	**Florencia**	6.27 ± 0.256 a	3.35 ± 0.097 a	176.01 ± 7.892 a	314.21 ± 2.02 a	32.66 ± 0.062 b	0.24 ± 0.004 a	109.02 ± 1.628 a	0.833 ± 0.002 a	0.83 ± 0.002 a	0.80 ± 0.008 a	0.02 ± 0.004 a
	**San Vicente**	6.21 ± 0.256 a	3.22 ± 0.097 a	169.34 ± 7.892 a	299.50 ± 2.02 b	32.14 ± 0.062 c	0.19 ± 0.004 c	86.13 ± 1.628 c	0.782 ± 0.002 c	0.78 ± 0.002 c	0.76 ± 0.008 b	0.02 ± 0.004 a
**Clone**	**CDC 312**	6.10 ± 0.289 a	3.08 ± 0.112 a	146.72 ± 9.577 b	302.43 ± 3.14 b	32.76 ± 0.096 d	0.23 ± 0.006 a	105.08 ± 2.641 a	0.816 ± 0.004 ab	0.81 ± 0.003 a	0.78 ± 0.012 a	0.02 ± 0.006 b
	**CDC 56**	5.41 ± 0.289 b	3.12 ± 0.112 a	166.49 ± 9.577 b	311.84 ± 3.14 a	32.22 ± 0.096 e	0.22 ± 0.006 a	100.11 ± 2.641 a	0.809 ± 0.001 b	0.81 ± 0.003 a	0.78 ± 0.012 a	0.02 ± 0.006 b
	**FDR 4575**	5.97 ± 0.289 a	3.11 ± 0.112 a	155.24 ± 9.577 b	314.95 ± 3.14 a	33.08 ± 0.096 c	0.23 ± 0.006 a	102.68 ± 2.641 a	0.816 ± 0.003 ab	0.81 ± 0.003 a	0.78 ± 0.012 a	0.02 ± 0.006 b
	**FDR 5597**	4.43 ± 0.289 c	2.67 ± 0.112 b	117.68 ± 9.577 c	314.03 ± 3.14 a	33.92 ± 0.096 a	0.22 ± 0.006 a	99.78 ± 2.641 a	0.812 ± 0.003 ab	0.81 ± 0.003 a	0.81 ± 0.012 a	0.02 ± 0.006 b
	**FDR 5788**	6.39 ± 0.289 a	3.29 ± 0.112 a	202.80 ± 9.577 a	317.05 ± 3.14 a	31.59 ± 0.096 f	0.20 ± 0.006 b	91.00 ± 2.641 b	0.814 ± 0.002 ab	0.81 ± 0.003 a	0.76 ± 0.012 a	0.02 ± 0.006 b
	**FX 3899 P1**	3.36 ± 0.289 d	2.25 ± 0.112 c	101.13 ± 9.577 c	318.97 ± 3.14 a	33.53 ± 0.096 b	0.20 ± 0.006 b	87.64 ± 2.641 b	0.814 ± 0.002 ab	0.81 ± 0.003 a	0.79 ± 0.012 a	0.01 ± 0.006 b
	**FX 4098**	6.27 ± 0.289 a	3.46 ± 0.112 a	186.90 ± 9.577 a	310.20 ± 3.14 a	32.11 ± 0.096 e	0.22 ± 0.006 a	97.00 ± 2.641 a	0.815 ± 0.002 ab	0.81 ± 0.003 a	0.77 ± 0.012 a	0.02 ± 0.006 b
	**GU 198**	6.34 ± 0.289 a	3.20 ± 0.112 a	164.68 ± 9.577 b	302.43 ± 3.14 b	32.30 ± 0.096 e	0.22 ± 0.006 a	99.95 ± 2.641 a	0.817 ± 0.003 a	0.81 ± 0.003 a	0.80 ± 0.012 a	0.02 ± 0.006 b
	**IAN 873**	6.24 ± 0.289 a	3.23 ± 0.112 a	173.61 ± 9.577 b	309.35 ± 3.14 a	32.47 ± 0.096e	0.23 ± 0.006 a	103.26 ± 2.641 a	0.808 ± 0.004 b	0.80 ± 0.003 a	0.77 ± 0.012 a	0.02 ± 0.006 b
	**MDF 180**	5.06 ± 0.289 b	2.73 ± 0.112 b	124.53 ± 9.577 c	307.21 ± 3.14 a	32.97 ± 0.096 c	0.22 ± 0.006 a	97.47 ± 2.641 a	0.812 ± 0.004 ab	0.81 ± 0.003 a	0.75 ± 0.012 a	0.04 ± 0.006 a
**Hour**	**6:00**	5.46 ± 0.200 c	3.24 ± 0.077 b	212.15 ± 6.253 a	324.42 ± 2.26 b	29.30 ± 0.069 e	0.19 ± 0.003 c	85.04 ± 1.508 c	-	-	-	-
	**9:00**	8.27 ± 0.200 a	3.72 ± 0.077 a	195.30 ± 6.253 b	287.12 ± 2.26 c	33.13 ± 0.069 c	0.28 ± 0.003 a	126.12 ± 1.508 a	-	-	-	-
	**12:00**	7.96 ± 0.200 a	3.68 ± 0.077 a	159.17 ± 6.253 c	273.36 ± 2.26 d	35.09 ± 0.069 a	0.28 ± 0.003 a	126.24 ± 1.508 a	-	-	-	-
	**15:00**	6.04 ± 0.200 b	2.88 ± 0.077 c	123.04 ± 6.253 d	278.18 ± 2.26 d	34.30 ± 0.069 b	0.24 ± 0.003 b	107.90 ± 1.508 b	-	-	-	-
	**18:00**	0.06 ± 0.200 d	1.55 ± 0.077 d	80.24 ± 6.253 e	390.33 ± 2.26 a	31.66 ± 0.069 d	0.10 ± 0.003 d	46.69 ± 1.508 d	-	-	-	-

^a^Standard error

^b^Means in each column followed by the same letter not differ statistically (Fisher’s LSD test, *p* < 0.05)

- Does not apply

Overall, the means of *A*, *E*, *g*_*s*_, *Φ*_PSII_ and *ETR* were significantly higher in the clones CDC 312, FDR 4575, FDR 5788, FX 4098, GU 198 and the IAN 873 control (Table 4). The clones with lower averages in parameters *A*, *E* and *g*_*s*_ were FX 3899 P1 and FDR 5597. At 9:00 and 12:00 h, the highest averages of A, E, Φ_PSII_ and ETR were seen (Table 4). The means of *g*_*s*_ and *C*_*i*_ were significantly higher at 6:00 h. At 12:00 h the highest average for *LT* was observed.

**Fig 2:**
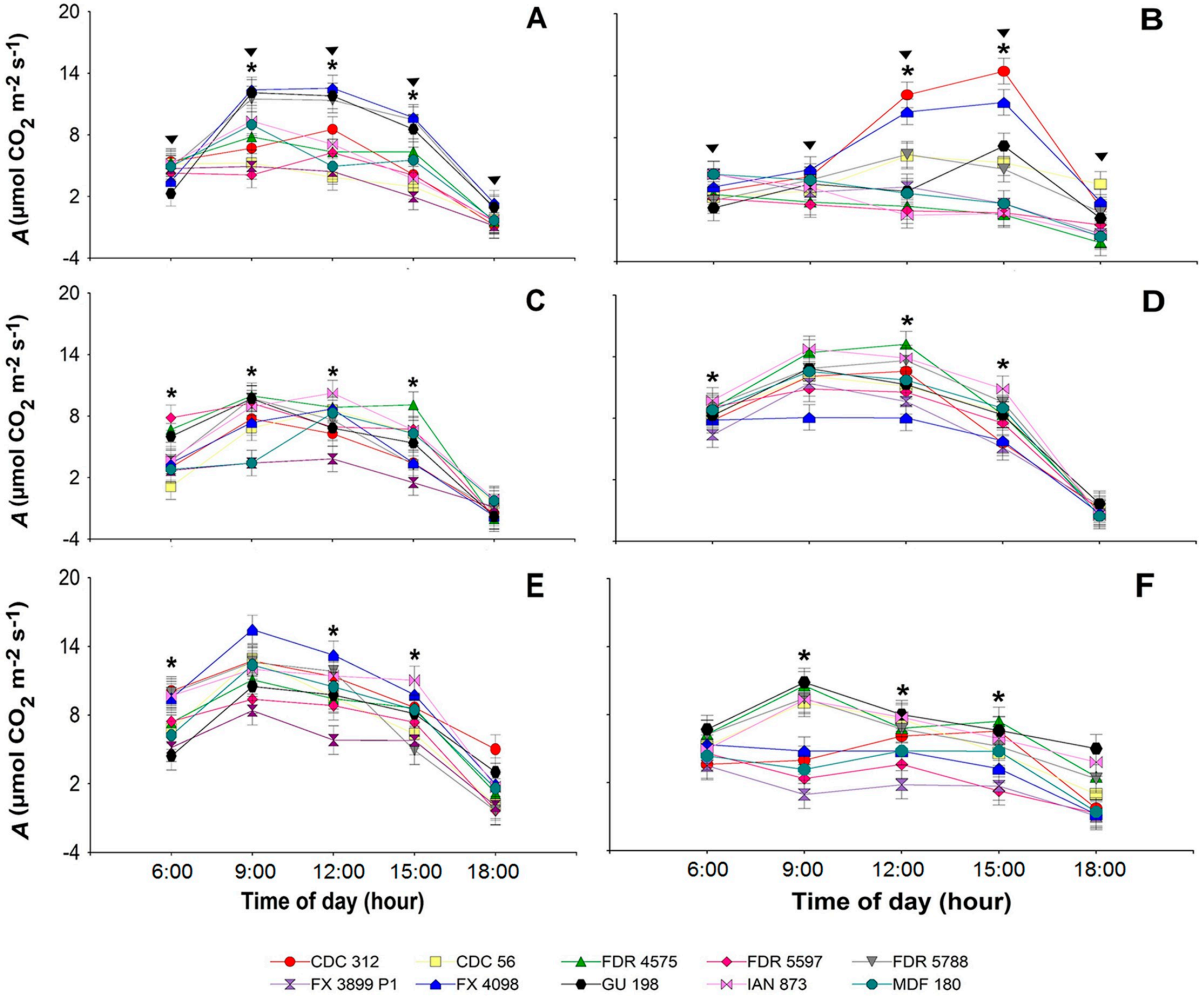
Daily net CO_2_ assimilation rate (*A*) in 10 rubber tree (*Hevea brasiliensis*) clones under two climatic periods at three sites in Caquetá (Colombia). (A), (C) and (E), dry period; (B), (D) and (F), rainy period; (A) and (B), Belén de los Andaquíes; (C) and (D), Florencia; (E) and (F), San Vicente del Caguán. Means for the dry and rainy periods followed by an inverted triangle and for the clones followed by an asterisk (*) for each time of day were significantly different according to Fisher’s LSD test, (*p* < 0.05). Bars represent the standard error of the mean; *n* = 4.

As shown in Fig 2, *A* showed an increase between 9:00 and 12:00 h, followed by a progressive decline towards sunset. This pattern only differed in clones CDC 312, FX 4098 and GU 198 in Belén de los Andaquíes in the rainy season, where the peaks occurred between 12:00 and 15:00 h (Fig 2B). In the rainy period in San Vicente del Caguán, it was observed that clones FX 4098, CDC 312, MDF 180, FDR 5597 and FX 3899 P1 recorded the lowest *A* average at 9:00 h (Fig 2F).

**Fig 3:**
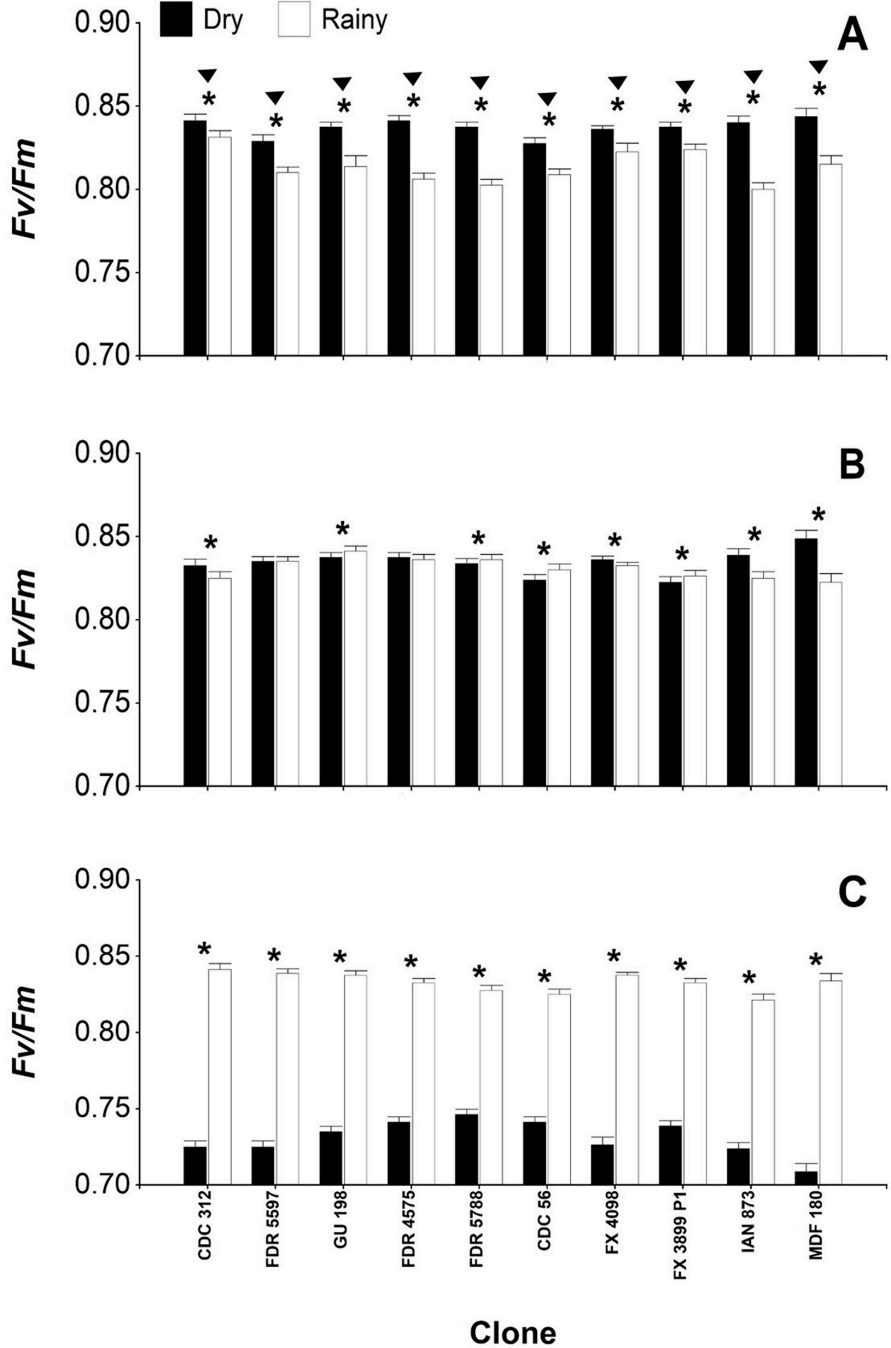
Maximum photochemical efficiency of PSII (*F*_*v*_*/F*_*m*_) in 10 rubber tree (*Hevea brasiliensis*) clones under two climatic periods at three sites in Caquetá (Colombia). Measurements carried out in leaves adapted to darkness (predawn, 3:00 h). **(**A), Belén de los Andaquíes; (B), Florencia; (C), San Vicente del Caguán. Means for the Belén de los Andaquíes, Florencia and San Vicente del Caguán sites followed by an inverted triangle and for the dry and rainy periods followed by an asterisk (*) for each clone were significantly different according to Fisher’s LSD test, (*p* < 0.05). Bars represent the standard error of the mean; *n* = 4.

According to Fig 3, independent of the period, all rubber clones had the highest average *F*_*v*_*/F*_*m*_ (0.82–0.84) in Belén de los Andaquíes and Florencia (Fig 3A and 3B), as compared with San Vicente del Caguán (0.77–0.79) (Fig 3C). When including the effect of the period, it was observed that most of the clones had the highest average of *F*_*v*_*/F*_*m*_ (0.82–0.84) in the dry period of Belén de los Andaquíes and Florencia. However, in the rainy period of San Vicente del Caguán, all clones presented means significantly similar to those observed in the other two sites.

### Pearson correlation

The Pearson's correlation analysis showed a positive correlation between *A* and the parameters *E* and *g*_*s*_ in both periods, and between *E* and *g*_*s*_, while the correlation of *C*_*i*_ with these parameters was negative (except with *E* in the dry period) (Table 5). *LT* presented a negative correlation with *A*, *E* and *g*_*s*_ in both periods and showed a positive correlation with *Φ*_PSII_, *ETR*, *F*_*v*_*/F*_*m*_ and *F*_*v*_*'/F*_*m*_*'* in the dry period, but a negative correlation in the rainy season with *Φ*_PSII_ and *ETR* and a positive correlation in both periods with *C*_*i*_ (Table 5). *Φ*_PSII_ and *ETR* had a positive correlation with each other and with the parameters *F*_*v*_*/F*_*m*_ and *F*_*v*_*'/F*_*m*_*'* in the two periods (Table 5). In the dry period, *A* and *E* presented a negative correlation with *Φ*_PSII_, *ETR*, *F*_*v*_*/F*_*m*_ and *F*_*v*_*'/F*_*m*_*'* (Table 5).

**Table 5:** Pearson's correlation coefficients for leaf gas exchange and fluorescence parameters of chlorophyll *a* measured in 10 rubber tree (*Hevea brasiliensis*) clones in the Colombian Amazon. Dry period (below the diagonal) and rainy period (above the diagonal).

Parameters	*A*	*E*	*g*_*s*_	*Ci*	*LT*	*Φ*_PSII_	*ETR*	*F*_*v/*_*F*_*m*_	*F*_*v*_*'/F*_*m*_*'*	*qP*	*NPQ*
***A***	…	0.92 **	0.85 **	-0.53 **	-0.61 **	0.66 **	0.67 **	0.33 **	0.33 **	0.04 ns	0.00 ns
***E***	0.89 **	…	0.95 **	-0.37 **	-0.67 **	0.61 **	0.61 **	0.30 **	0.30 **	-0.01 ns	0.01 ns
***g***_***s***_	0.83 **	0.93 **	…	-0.23 **	-0.73 **	0.60 **	0.60 **	0.23**	0.22 **	-0.07 ns	0.03 ns
***Ci***	-0.53 **	0.93 **	-0.12ns	…	0.18 *	-0.47 **	-0.47 **	-0.50 **	-0.48 **	-0.01 ns	-0.06 ns
***LT***	-0.61 **	-0.63 **	-0.72 **	0.21 **	…	-0.44 **	-0.43 **	-0.17 ns	-0.16 ns	-0.01 ns	-0.05 ns
***Φ***_**PSII**_	-0.25 **	-0.19 *	-0.12 ns	0.42 **	0.43 **	…	1.00 **	0.34 **	0.34 **	-0.08 ns	0.00 ns
**ETR**	-0.25 **	-0.19 *	-0.12 ns	0.42 **	0.43 **	1.00 **	…	0.35 **	0.34 **	-0.09 ns	0.00 ns
***F***_***v***_***/F***_***m***_	-0.54 **	-0.46 **	-0.32 **	0.57 **	0.39 **	0.84 **	0.84 **	…	0.95 **	-0.05 ns	0.01 ns
***F***_***v***_***´/F***_***m***_***´***	-0.54 **	-0.47 **	-0.33 **	0.56 **	0.39 **	0.83 **	0.83 **	0.99 **	…	0.03 ns	-0.25 **
***qP***	0.03 ns	0.04 ns	0.01 ns	0.10 ns	0.09 ns	0.13 ns	0.13 ns	0.10 ns	0.09 ns	…	-0.35 **
**NPQ**	0.11 ns	0.20 *	0.17 ns	0.00 ns	-0.04 ns	0.08 ns	0.08 ns	-0.00 ns	-0.06 ns	0.14 ns	…

Net CO_2_ assimilation rate (*A*), transpiration rate (*E*), stomatal conductance (*g*_*s*_), intercellular CO_2_ concentration (*Ci*), leaf temperature (*LT*), photochemical efficiency of PSII (*Φ*_PSII_), electron transport rate (*ETR*), maximum photochemical efficiency of PSII (*F*_*v*_*/Fm*), efficiency of excitation energy captured by open PSII reaction centers (*F*_*v*_*'/F*_*m*_*'*), photochemical quenching coefficient (*qP*), non-photochemical quenching (*NPQ*).

*p < 0.05; significant

**p < 0.01, very significant

ns, not significant.

### Co-Inertia analysis

**Fig 4:**
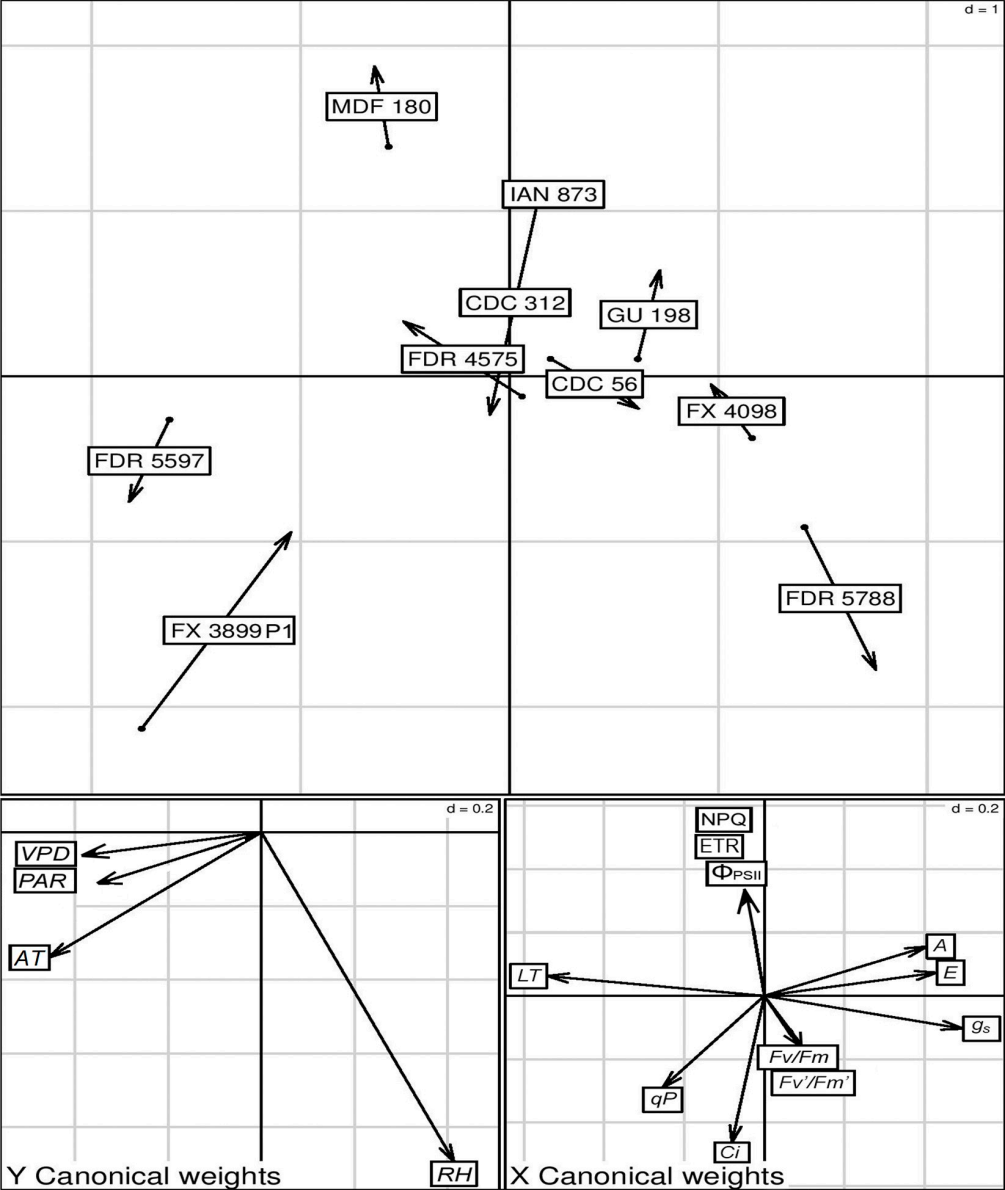
Results of the Co-Inertia analysis between the foliar micro-environmental parameters and photosynthesis variables in 10 *Hevea brasiliensis* clones. (A) a factorial co-Inertia plane for the clones. (B) and (C) the projection of the micro-environmental and photosynthesis vectors, respectively, in the factorial co-Inertia plane. The arrow head represents the position of the clones for the photosynthesis variables, and the other extreme shows the micro-environmental parameters. The bigger the arrow is, the less of a relationship there was between the micro-environmental parameters and photosynthesis.

The Montecarlo test of the Co-Inertia analysis was very significant (*p* < 0.01), which means the co-structure described by axes 1 and 2 was similar to the structures described in the individual analysis (principal component analysis) of each group of variables (micro-environmental and photosynthesis). The first two axes of the Co-Inertia analysis explained 98.52% and 0.92% of the total variability, which indicates that the studied variables were sufficient to explain the ordination observed in the evaluated clones (Fig 4).

According to Fig 4, the intensity of the relationship between the studied groups of variables was different in the different clones. The strongest relationship was observed in the clones with the highest gas exchange values and the lowest micro-environmental parameters. The clones with better photosynthetic performance (desirable genotypes: FDR 5788, GU 198 and FX 4098) were located to the right of the ordination axis 1, which demonstrated greater photosynthetic rates (*A*) and a better ability in the photosynthetic apparatus (PSII) to capture and use light energy (*F*_*v*_*/F*_*m*_, *F*_*v*_*'/F*_*m*_*'* and *qP*). The FX 3899 P1 and FDR 5597 clones were mainly grouped towards the left of the ordination axis 1, with the lowest photosynthetic performance and the highest values for the micro-environmental parameters (except the *RH*). The MDF 180 clone, with the best photosynthetic capacity to dissipate light energy (highest value of *NPQ*) (Table 4), was located on the positive extreme of axis 2 of the ordination plane.

## Discussion

Although the Amazon region has rainfall throughout the year, it also has seasonality in its precipitation and radiation [49]. In the present study, not only were there differences in the microclimatic parameters *PAR*, *AT*, *VPD* and *RH* between the dry and rainy periods, but there was also a significant variation in the physiological response between both periods for all the studied sites. According to Renninger and Phillips [49] and Zhang et al. [50], in the Amazon, environmental radiation is the most important limiting factor for the rubber plant since, in the rainy season, cloudiness is greater and the amount of light (‘sun flecks’) that can be used for photosynthesis is limited [51]. In the present study, this condition was evidenced by a lower *A* in the rainy season. However, in the rainy period, the *g*_*s*_ and *E* were higher, which favored stomatal opening as a result of higher *RH* values and lower values of *AT* and *VPD* [52]. Likewise, the positive correlation observed between *A* and *g*_*s*_ and the daytime pattern of these parameters in both periods suggest that both processes lead to a higher photosynthetic performance in the morning hours, where there is greater stomatal openings, favoring higher assimilation rates of CO_2_ [53]. Light is one of the most important environmental factors that influence both *A* and *g*_*s*_, and the variation in light energy received by the plant can create rapid and extreme fluctuations in leaf temperature and leaf–air vapour pressure deficit to which stomata will respond in conjunction with other environmental cues [54]. In this study, a decreasing pattern for *g*_*s*_ at midday in both seasons was observed. In citrus and many other species, stomata closure has been observed in plants around midday, the time when reached the highest *VPD* and temperature, which generates a reduction in stomata conductance (*g*_*s*_) and thus a characteristic decrease in CO_2_ assimilation [51].

Several studies have also reported an increase in photosynthesis in the dry period in the Amazon region because water is not a limiting factor, but light is [55–57]. Although the dry evapotranspiration period is greater because of an increase in radiation and temperature [49], a partial closure of the stomata would be expected because of a higher *VPD* [52,58] as compared with the rainy period, possibly because of the deep root system of rubber trees, combined with the hydraulic redistribution of the roots [59,60].

In the present study, the variations observed in the photosynthetic responses to light in the different rubber clones responded to the intraspecific genetic variability of this species, where these genotypes showed a greater photosynthetic capacity (higher values in *A*_*max*_, *LCP*, *LSP* y *R*_*d*_) with respect to other clones analyzed in similar studies [8,61].

These variations also showed the diurnal behavior of the clones in the different areas, where they evidenced a diurnal behavior for the net photosynthetic rate (*A*) in the different periods and sites, with GU 198 and FX 4098 recording higher *A* values between 9:00 and 12:00 h (higher *PAR*). These two clones, despite not reporting the highest *A*_*max*_, showed high efficiency in the quantum conversion (*A*_*qe*_), low light compensation points (*PCL*) and a higher *A*_*max*_ with a lower *PAR*, as compared with the other clones. According to Gunasekera et al. [61], in terms of increasing productivity in *H*. *brasiliensis*, a low *PCL* value is important since it keeps the photosynthetic rate (*A*) positive even when the light intensity is low and, therefore, continues the accumulation of dry matter under these conditions.

Clones FX 4098, CDC 312, FDR 4575, FDR 5788 and GU 198 recorded the highest average values of *A*, *E*, *gs*, *J* and *Φ*_*PSII*_ in both climatic periods during the diurnal cycle, reflecting a greater physiological capacity of the plants to maintain the photosynthetic apparatus and express better photosynthetic performance against changes and limitations of the underlying environmental conditions [62]. This behavior is useful for the selection of promising genotypes because clones that present a better adaptation in their photosynthetic apparatus to environmental stress factors will express higher growth rates and greater production potential in the field [10,20,26].

In rubber, photosynthetic rates and efficient water use are physiological variables that have been associated with high-performance clones [9]. Ahmad et al. [10] found that parameters such as *A*, *E*, *g*_*s*_ and stomata characteristics have a positive correlation with latex performance, making they important parameters for the selection of new clones.

The decrease in the *F*_*v*_*/F*_*m*_ and *F*_*v*_*'/F*_*m*_*'* in the dry period was possibly related to reversible changes in the electron transport flow and heat dissipation used by the photosynthetic apparatus to adjust the quantum efficiency of the PSII and, thus, avoid damage at the level of the photosynthetic system [58], which could explain the negative correlation of these parameters with *A* in the dry period. However, the values recorded for *F*_*v*_*/F*_*m*_ did not indicate photoinhibition in any of the sites or periods since the values were above 0.78 [40]. *Φ*_*PSII*_ is the proportion of absorbed energy being used in photochemistry, and *qP* gives an indication of the proportion of PSII reaction centers that are open [22]. In this study, *Φ*_*PSII*_, *J* and *qP* were higher in both seasons and three LSCT, indicating the rubber’s clones had a high photochemical capacity and a greater efficiency at transferring light energy from the light-harvesting complex to PSII. Also, *NPQ* indicates that high levels of light energy that could exceed photosynthetic capability will be transformed into thermal dissipation [63], were lower indicate that dissipation of light energy were used in photochemical processes.

## Conclusions

The results of the present study show a significant effect of the phenotypic variation of *H*. *brasiliensis* on the photosynthetic behavior of rubber, and this, in turn, is directly influenced by the daily and seasonal micro-environmental variations characteristic of the Colombian Amazon. The best photosynthetic performance was observed in the dry period, between 9:00 and 12:00 h, in Florencia and San Vicente del Caguán.

These results lead to the conclusion that the evaluation of the temporal dynamics of the parameters of gaseous exchange and fluorescence of chlorophyll *a* in the rubber clones analyzed in the present study identifies the unproductive phase of the rubber crop, the potential physiological adaptation of these genotypes in the face of different agro-climatic conditions in the Colombian Amazon and, therefore, highlights the greater production potential that these materials may express differentially in the final phase of the productive evaluation.

The clones FX 4098, FDR 4575, MDF 180, GU198 and FDR 5788 are the genotypes with the best photosynthetic performance and the best phenotypic plasticity in the different periods and locations that were studied. These desirable genotypes constitute a promising gene pool for expanding the genetic resources of rubber trees in the Colombian Amazon region.

## Supporting information

S1 File Daily microclimatic variation in study area (Caquetá, Colombia). (TXT)

S2 File Daily net CO_2_ assimilation rate (*A*) in 10 rubber tree (*Hevea brasiliensis*) clones under two climatic periods at three sites in Caquetá (Colombia). (TXT)

S3 File Maximum photochemical efficiency of PSII (*F_v_/F_m_*) in 10 rubber tree (*Hevea brasiliensis*) clones under two climatic periods at three sites in Caquetá (Colombia). (TXT)

S4 File Results of the Co-Inertia analysis between the foliar micro-environmental parameters and photosynthesis variables in 10 *Hevea brasiliensis* clones. (TXT)
